# Comparative transcriptome analysis implied a *ZEP* paralog was a key gene involved in carotenoid accumulation in yellow-fleshed sweetpotato

**DOI:** 10.1038/s41598-020-77293-7

**Published:** 2020-11-26

**Authors:** Keisuke Suematsu, Masaru Tanaka, Rie Kurata, Yumi Kai

**Affiliations:** Kyushu Okinawa Agricultural Research Centre, National Agriculture and Food Research Organisation, 6651-2 Yokoichi, Miyakonojo, Miyazaki 885-0091 Japan

**Keywords:** Plant breeding, Plant molecular biology, Transcriptomics

## Abstract

The mechanisms of carotenoid accumulation in yellow-fleshed sweetpotato cultivars are unclear. In this study, we compared the transcriptome profiles of a yellow-fleshed cultivar, Beniharuka (BH) and two of its spontaneous white-fleshed mutants (WH2 and WH3) to reveal the genes involved in yellow flesh. As a result of RNA sequencing, a total of 185 differentially expressed genes (DEGs) were commonly detected in WH2 and WH3 compared to BH. Of these genes, 85 DEGs and 100 DEGs were commonly upregulated and downregulated in WH2 and WH3 compared to BH, respectively. g1103.t1, a paralog of *zeaxanthin epoxidase* (*ZEP*), was only DEG common to WH2 and WH3 among 38 genes considered to be involved in carotenoid biosynthesis in storage roots. The expression level of g1103.t1 was also considerably lower in five white-fleshed cultivars than in five yellow-fleshed cultivars. Analysis of carotenoid composition in the storage roots showed that the epoxidised carotenoids were drastically reduced in both WH2 and WH3. Therefore, we propose that the *ZEP* paralog, g1103.t1, may be involved in carotenoid accumulation through the epoxidation of β-carotene and β-cryptoxanthin in sweetpotato.

## Introduction

The flesh colour of the storage roots of the sweetpotato *Ipomoea batatas* (L.) Lam is one of the most important targets in breeding programs. Sweetpotato cultivars show various flesh colours such as white, yellow, orange and purple depending on their carotenoid or anthocyanin components. In Asia, especially in Japan, yellow-fleshed cultivars are popular for table use and processed products^1^. In addition to the commercial value of their appearance, yellow-fleshed cultivars may have healthcare functions. Some plant carotenoids have antioxidant activity and are thought to contribute to preventing cancer, heart disease and eye diseases^2,3^, and carotenoids in yellow-fleshed sweetpotato have been shown to have such activity^4,5^.

**Figure 1:**
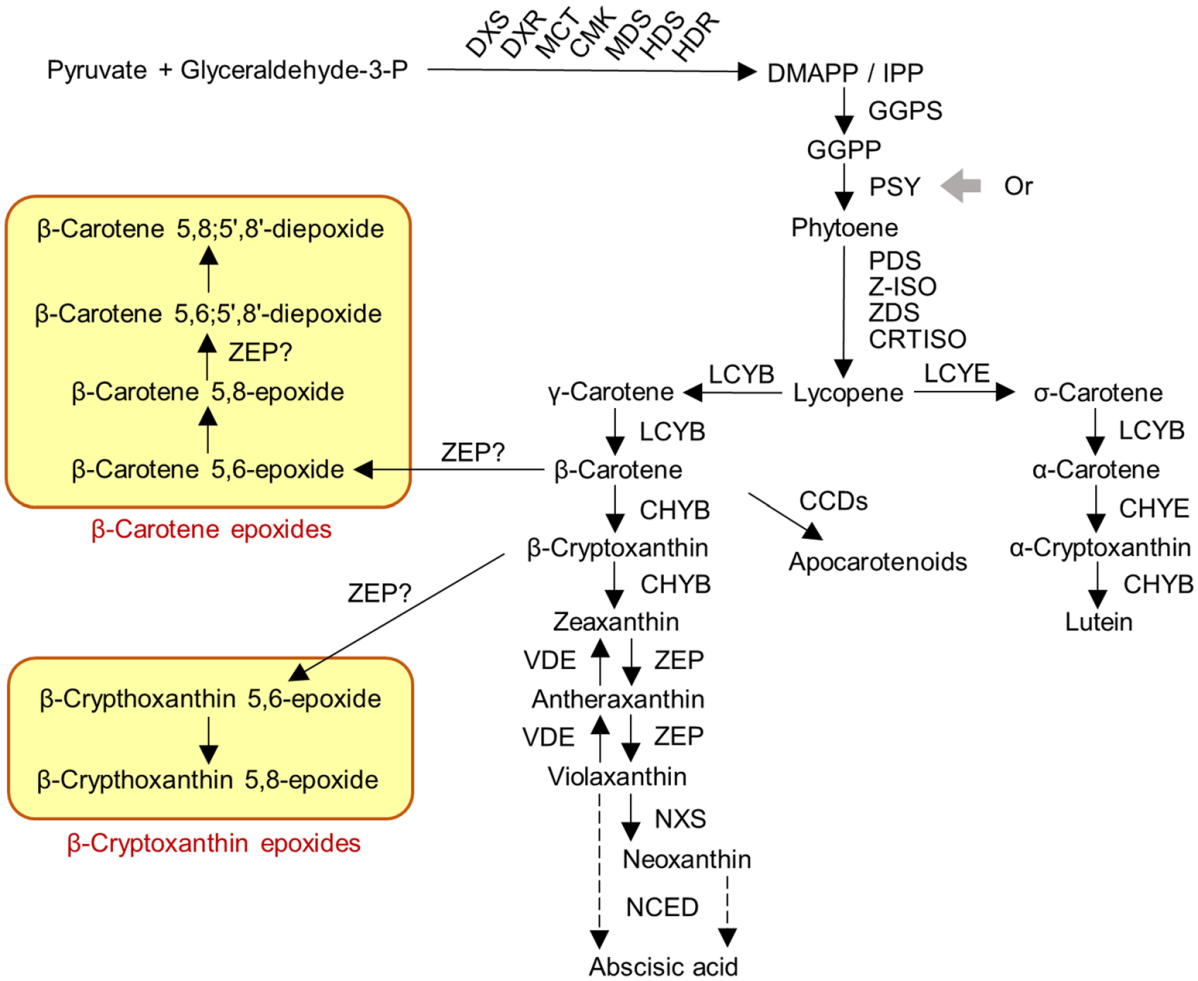
Proposed carotenoid biosynthetic pathway in sweetpotato. *DXS* 1-deoxy-d-xylulose 5-phosphate synthase, *DXR* 1-deoxy-d-xylulose 5-phosphate reductoisomerase, *MCT* 2-C-methyl-d-erythritol 4-phosphate cytidylyltransferase, *CMK* 4-(cytidine 5′-diphospho)-2-C-methyl-d-erythritol kinase, *MDS* 2-C-methyl-d-erythritol 2,4-cyclodiphosphate synthase, *HDS* 4-hydroxy-3-methylbut-2-enyl diphosphate synthase, *HDR* 4-hydroxy-3-methylbut-2-enyl diphosphate reductase, *DMAPP* dimethylallyl diphosphate, *IPP* isopentenyl pyrophosphate isomerase, *GGPS* geranylgeranyl pyrophosphate synthase, *GGPP* geranyl geranyl diphosphate, *PSY* phytoene synthase, *Or* orange, *PDS* phytoene desaturase, *Z-ISO* 15-cis-ζ-carotene isomerase, *ZDS* ζ-carotene desaturase, *CRTISO* carotenoid isomerase, *LCYB* lycopene β-cyclase, *LCYE* lycopene ε-cyclase, *CHYB* carotenoid β-hydroxylase, *CHYE* carotenoid ε-hydroxylase, *CCD* carotenoid cleavage dioxygenase, *ZEP* zeaxanthin epoxidase, *VDE* violaxanthin de-epoxidase, *NXS* neoxanthin synthase, *NCED* 9-cis-epoxycarotenoid dioxygenase.

Yellow-fleshed sweetpotato mainly contains unique carotenoids in the form of β-cryptoxanthin epoxides and β-carotene epoxides such as β-cryptoxanthin 5,8-epoxide, β-carotene 5,8;5′,8′-diepoxide, etc.^4,6^. These pigments cause a yellow colour in storage roots. Figure 1 shows a proposed biosynthetic pathway of carotenoids in sweetpotato^7–9^. A lot of genes regulate the biosynthesis of carotenoids. The flesh colour (white or yellow) of sweetpotato could be determined by the balance of the synthesis and metabolisation of these yellow pigments^1,5^. Recently, genes related to carotenoid accumulation were identified in sweetpotato and their functions were revealed^8^. The carotenoid content in sweetpotato calli was found to be increased by suppressing the gene expression of *IbLCYE*, *IbLCYB* and *IbCHYB* individually^10–12^, and overexpression of *IbOr* also increased carotenoid content in calli and storage roots^13–15^. However, the molecular mechanisms involved in the biosynthesis of β-cryptoxanthin epoxides and β-carotene epoxides remain unclear.

In the carotenoid metabolism of plants, the cyclisation of lycopene is an essential step^16^. β-Carotene is synthesised through the addition of β-rings to lycopene by lycopene β-cyclase (LCYB) (Fig. 1). Subsequent hydroxylation of β-carotene β-rings leads to the generation of zeaxanthin via β-cryptoxanthin. Zeaxanthin epoxidase (ZEP) then catalyses the epoxidation of β-rings in zeaxanthin and antheraxanthin. On the other hand, β-cryptoxanthin epoxides and β-carotene epoxides in yellow-fleshed sweetpotato are generated by the epoxidation of β-rings in β-cryptoxanthin and β-carotene, respectively. Khan et al. (2016)^7^ suggest that these epoxidation steps may be catalysed by a novel enzyme highly homologous to ZEP. However, *ZEP* paralogs encoding such an enzyme have not yet been identified in sweetpotato.

Flesh-coloured mutants of various sweetpotato cultivars have contributed to revealing the genetic and physiological mechanisms underlying the pigmentation of storage roots. Tanaka et al. (2012)^17^ studied the gene structure and function of *IbMYB1* genes using a white-fleshed mutant (AYM96) of a purple-fleshed cultivar, Ayamurasaki. McGregor and LaBonte (2006)^18^ compared gene expression for β-carotene accumulation between Jewel and its mutant (White Jewel). Recently, several spontaneous white-fleshed mutants of the cultivar Beniharuka (BH), whose flesh colour is pale yellow, were found. BH and its mutant series would be useful in an exploration of the accumulation mechanism of yellow pigments like β-cryptoxanthin epoxides and β-carotene epoxides.

Sweetpotato has a complex genome with hexaploidy (2n = 6x = 90) and high heterozygosity, and little is known about its genome or genes. Recently, the draft genome sequences of sweetpotato^19^ and *I. trifida*, a diploid wild relative of sweetpotato^20,21^ were released and became available for researchers and breeders. These databases make genomic and transcriptomic approaches easier in studies on sweetpotato. Transcriptome sequencing using a next-generation sequencer is effective to analyse the gene expression profile involved in flesh colour. RNA sequencing (RNA-seq) by de novo assembly has revealed the expression of genes related to anthocyanin accumulation in purple-fleshed sweetpotato cultivars^22,23^ and β-carotene accumulation in orange-fleshed cultivars^24,25^. Gemenet et al. (2020)^26^ also revealed that the *phytoene synthase* gene (*PSY*) played an important role in the carotenoid accumulation in orange-fleshed storage roots by combining transcriptome analysis and QTL analysis. However, it remains unknown what genes are essential for the accumulation of carotenoids in yellow-fleshed sweetpotato cultivars. In the present study, we used a yellow-fleshed cultivar (BH) and its two white-fleshed mutants (WH2 and WH3) and compared the gene expression profiles in their storage roots using Illumina paired-end sequencing technology to identify the genes involved in yellow flesh.

## Results

### Characteristics of BH, WH2 and WH3

**Figure 2:**
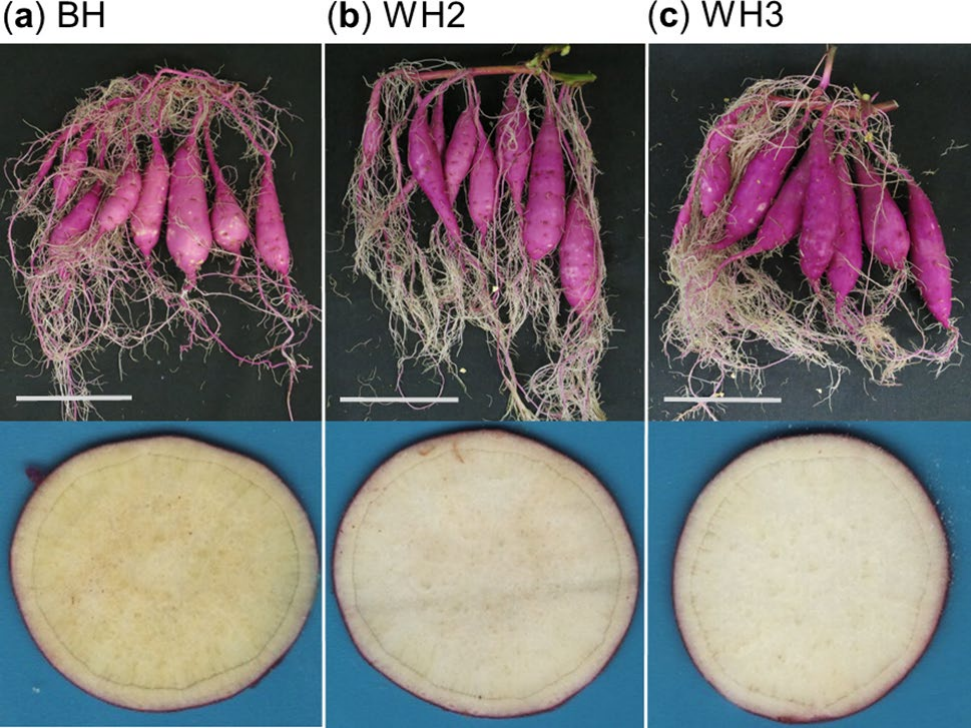
Phenotypic characteristic appearance and cross sections of the storage roots of (**a**) the Beniharuka cultivar (BH), and its two white-fleshed mutants (**b**) WH2 and (**c**) WH3. Bars = 10 cm.

The storage roots of BH, WH2 and WH3 used in this experiment are shown in Fig. 2. The a* (red/green) and b* (yellow/blue) values indicated a significant difference in the flesh colours of BH, WH2 and WH3 (Supplementary Table S1) in accordance with their visible appearance. The a* value of BH was lower than those of WH2 and WH3, and its b* value was higher. Measured traits other than flesh colour did not differ among these lines (Supplementary Table S1).

**Figure 3:**
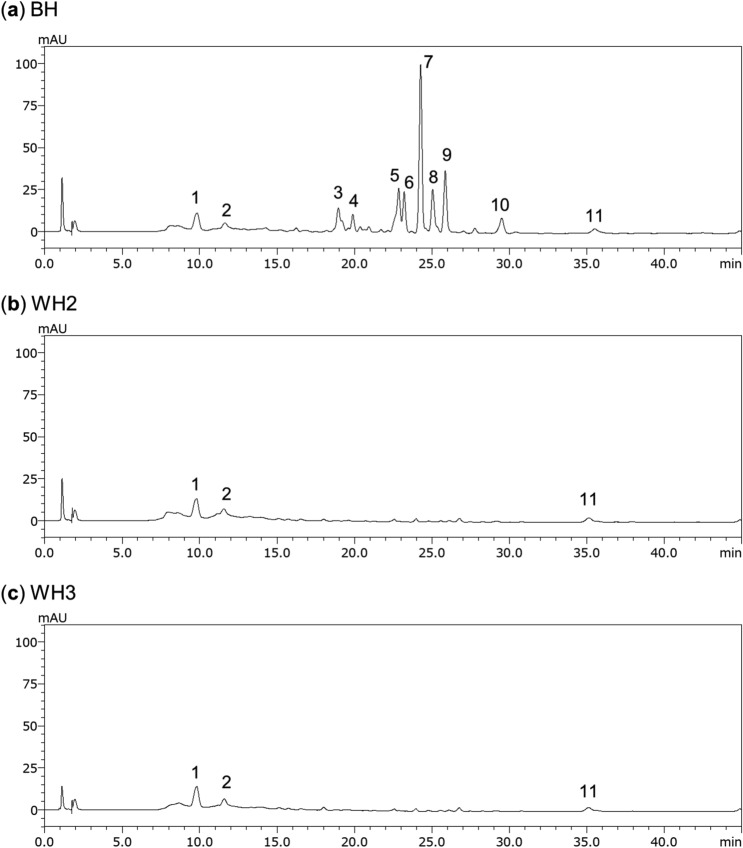
Chromatograms of carotenoids extracted from (**a**) BH, (**b**) WH2 and (**c**) WH3. Peaks were identified by comparing with Ishiguro et al. (2010)^4^: unknown (1), unknown (2), unknown (3), ipomoeaxanthin C1 (4), β-cryptoxanthin 5,8-epoxide (5), unknown (6), β-carotene 5,8;5′,8′-diepoxide (*cis*-isomer) (7), β-carotene 5,8;5′,8′-diepoxide (diastereomer) (8, 9), β-carotene 5,8-epoxide (10), β-carotene (11).

The total carotenoid contents in WH2 and WH3 estimated from the total peak area detected by high performance liquid chromatography (HPLC) chromatograms were more than 85% lower than that in BH (Fig. 3, Supplementary Table S2). Also, the peaks corresponding to the major carotenoids in BH, β-carotene-5,8;5′,8′-diepoxide and β-cryptoxanthin 5,8-epoxide (Fig. 3a, Supplementary Table S2), were not detected in WH2 or WH3 (Figs. 3b,c, Supplementary Table S2). These results suggest that the white flesh of WH2 and WH3 is caused by this large decrease in carotenoids.

### Transcriptome profiling in BH, WH2 and WH3 storage roots

RNA-seq data of BH, WH2 and WH3 storage roots with three biological replicates were generated using Illumina NovaSeq 6000. On average, 44 million reads were obtained after removing low-quality reads and Illumina adaptor sequences. Of these reads, approximately 90% were mapped to the reference genome using HISAT2.

**Figure 4:**
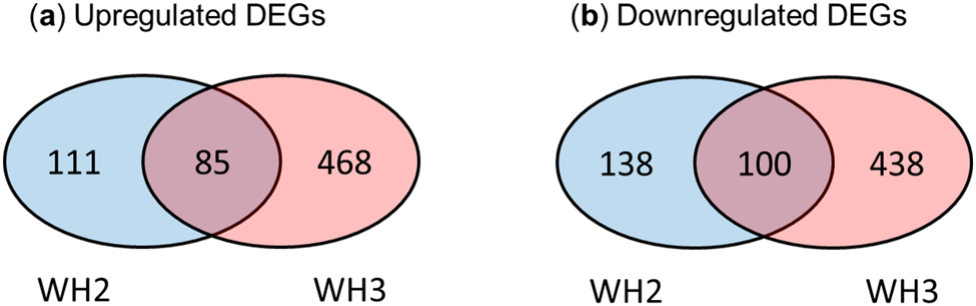
Venn diagrams showing the number of differentially expressed genes (DEGs) identified by RNA-seq analysis. (**a**) Number of upregulated genes in WH2 and WH3 compared to BH. (**b**) Number of downregulated genes in WH2 and WH3 compared to BH.

To identify differentially expressed genes (DEGs) in WH2 and WH3, gene expression was compared between BH and WH2, and between BH and WH3 (Supplementary Fig. S1) using featureCounts and edgeR. Totals of 249 and 906 DEGs in WH2 and WH3, respectively, were detected after filtering with |log2 fold changes (logFC)|≥ 2, average log2-counts-per-million (logCPM) ≥ 1 and false discovery rate (FDR) ≤ 0.01. Of these, 111 DEGs in WH2 and 468 in WH3 were upregulated compared to BH (Fig. 4a**,** Supplementary Table S3 and S4). Among the upregulated DEGs, 85 were common to WH2 and WH3 (Supplementary Table S5). In contrast, 138 DEGs in WH2 and 438 in WH3 were downregulated compared to BH (Fig. 4b**,** Supplementary Table S6 and S7). Among the downregulated DEGs, 100 were common to WH2 and WH3 (Supplementary Table S8).

To estimate the function of DEGs, transcript sequences obtained from the Ipomoea Genome Hub website (https://ipomoea-genome.org/) were annotated by sequence-similarity searches against the UniProtKB database using Hayai-Annotation Plants. Out of the total 64,295 predicted genes, 38,788 (60.3%) genes were matched to sequences in UniProtKB with a sequence identity > 40%, query coverage > 40% and e-value < 10^–6^. In addition, 19,272 (49.7%), 32,095 (82.7%) and 30,017 (77.4%) genes of the annotated 38,788 genes were assigned to gene ontology (GO) terms in the biological process, cellular component and molecular function, respectively. Of the DEGs of WH2 and WH3, 211 and 785 genes were assigned to GO terms, respectively, and further classified into 44 GO term categories using WEGO2 software (Supplementary Fig. S2). Although the numbers of DEGs were different between WH2 and WH3, the percentage of DEGs classified to each category were similar between them. In the cellular component category, most WH2 and WH3 DEGs were classified to cell, organelle, membrane and membrane part. In the molecular function category, DEGs in WH2 and WH3 were largely in catalytic activity and binding. In the biological process category, cellular process and metabolic process were representative classes of WH2 and WH3 DEGs. This similarity between DEGs in WH2 and WH3 suggests that WH2 and WH3 share the molecular mechanism of absence of carotenoids.

### DEGs in carotenoid biosynthesis

**Figure 5:**
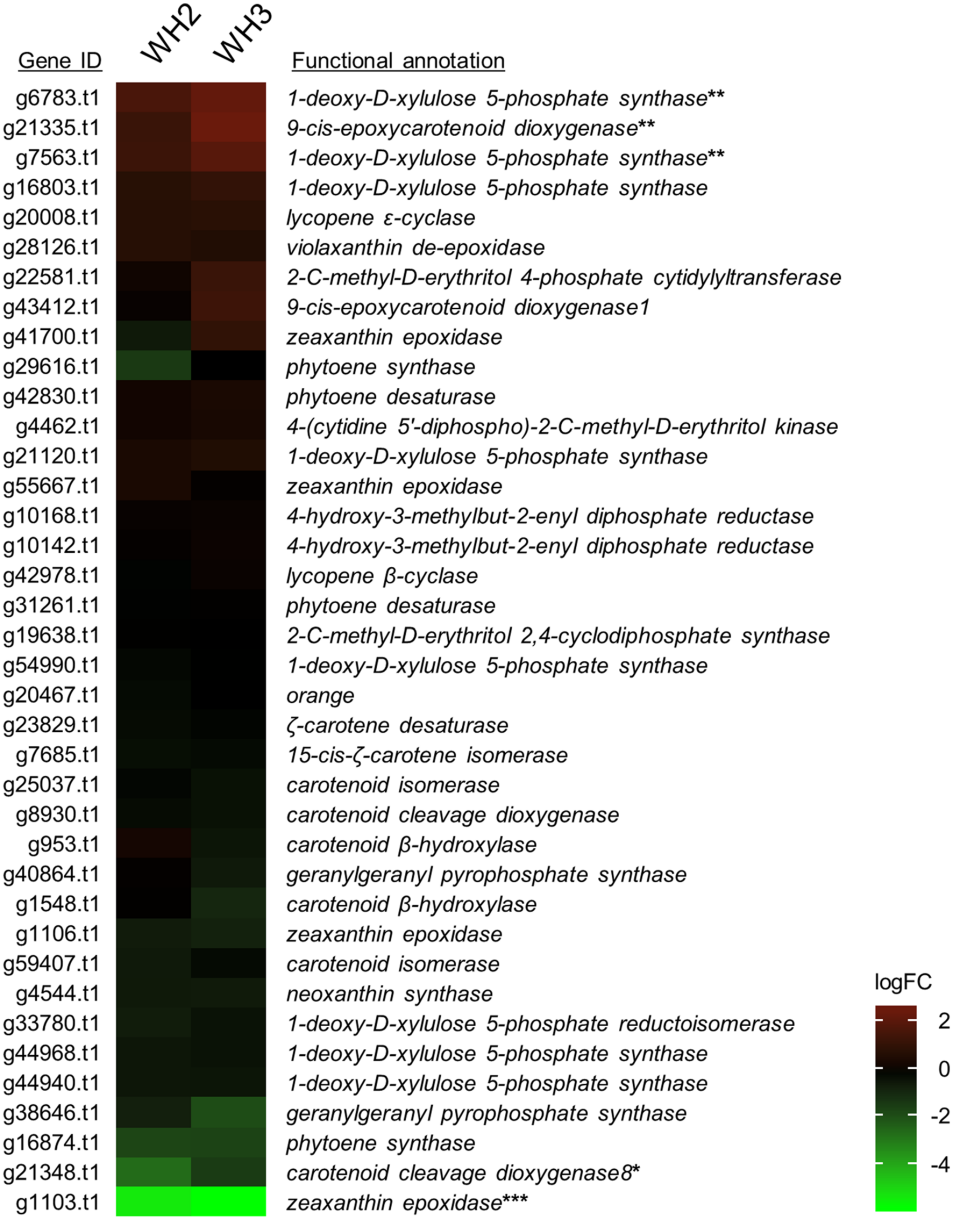
Heatmap of log2 fold change (logFC) values for the 38 genes related to carotenoid biosynthesis. The functional annotation of each gene is described in Fig. 1. *, ** and *** indicate DEGs in WH2, WH3 and both WH2 and WH3, respectively.

In order to reveal the genes responsible for the white-flesh mutation, we focused on previously known genes for carotenoid biosynthesis. Based on the functional annotation of UniProtKB and GO terms, 69 genes were identified as being involved in the carotenoid biosynthesis pathway shown in Fig. 1. Although g20008.t1 was not annotated by Hayai-Annotation Plants, its sequence showed high similarity to the *lycopene ϵ-cyclase* gene (*IbLCYE*, GenBank: HQ828093.1) of sweetpotato. Of these 70 genes including g20008.t1, 38 showed an average logCPM larger than 1 and are considered to constitute the expressed gene set of carotenoid biosynthesis in sweetpotato storage roots. In this gene set, only g1103.t1 (*ZEP*) was counted as a common DEG for both WH2 and WH3, showing logFC values of -5.33 and -5.76, respectively. Although g16874.t1 (*PSY*) was significantly downregulated in both WH2 and WH3 (Fig. 5), it was not counted as DEG, because its |logFC| values were less than 2. A few DEGs for either WH2 or WH3 were also observed: g21348 (*CCD8*) was counted as a DEG for only WH2, while g6783.t1 (*DXS*), g21335.t1 (*NCED*) and g7563.t1 (*DXS*) were DEGs for only WH3. Gene expression of g1103.t1 was extremely suppressed in both WH2 and WH3 in all DEGs (Supplementary Table S8). Thus, g1103.t1 was assumed to be one of the most promising candidate genes for yellow flesh, and we therefore conducted further characterization of this gene.

### Phylogenetic analysis of *ZEP*

**Figure 6:**
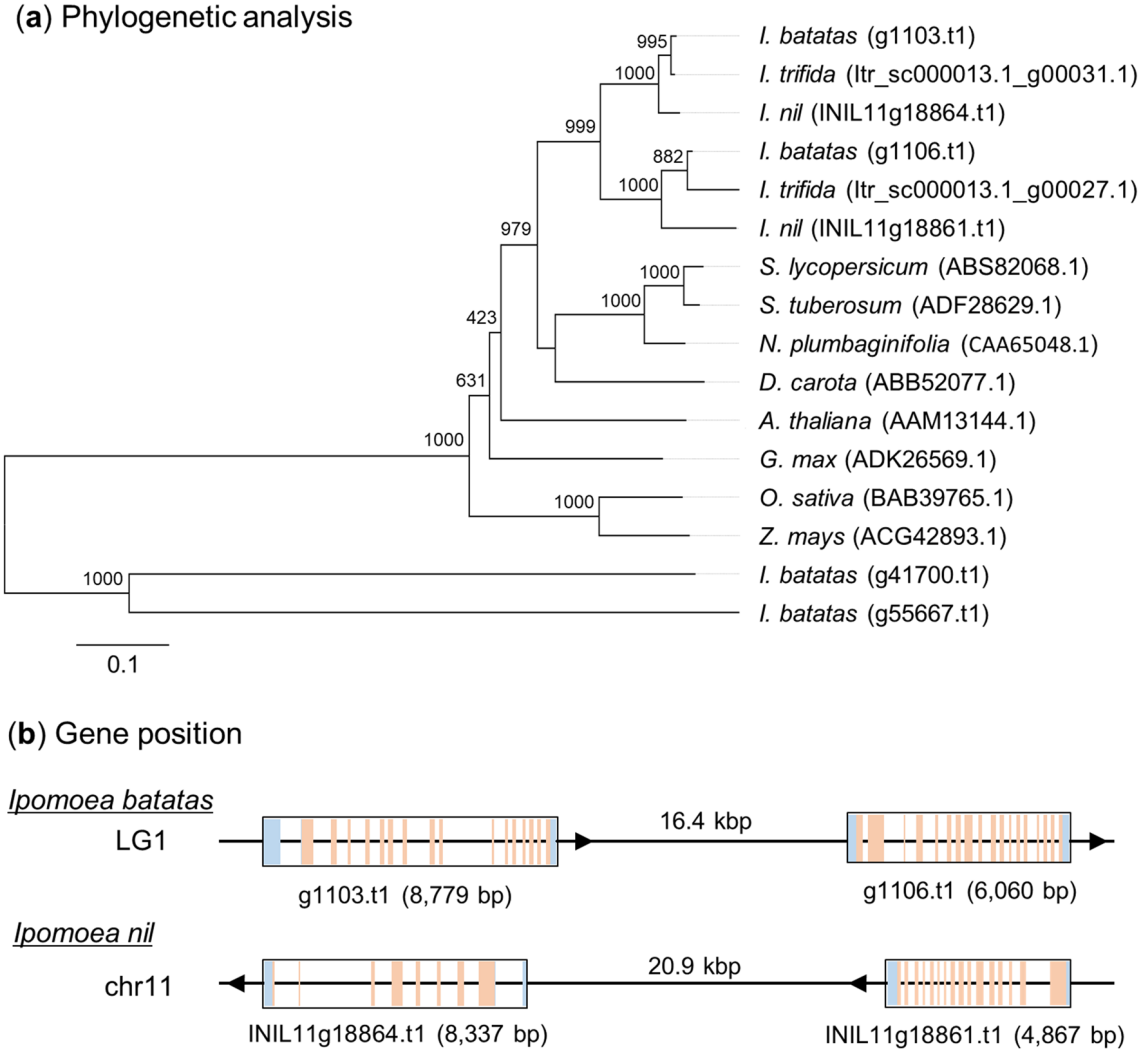
Characterisation of *zeaxanthin epoxidase* (*ZEP)* homologs. (**a**) Phylogenetic analysis of the deduced amino acid sequences of g1103.t1 and g1106.t1 with 1,000 bootstrap replications. The deduced amino acid sequences of *ZEP* homologs in *Ipomoea trifida* and *Ipomoea nil* were added to this analysis. ZEPs of eight additional species obtained from the National Centre for Biotechnology Information (NCBI) database are also incorporated into this analysis. The scale bar of 0.1 refers to 10% sequence divergence. (**b**) The genomic position of *ZEP* homologs in *I. batatas* and *I. nil*. Blue boxes indicate untranslated regions (UTRs) and orange boxes indicate exons in each gene. Arrows show the coding direction of each gene.

In the set of genes expressed in carotenoid biosynthesis, g1103.t1, g1106.t1, g41700.t1 and g55667.t1 were annotated as *ZEP* (Fig. 5). Of these, g1103.t1 and g1106.t1 are closely related to known functional *ZEP* genes in other plants. Although each *ZEP* gene was found in other plant species, sweetpotato had two *ZEP* homologues. Sequence similarity searches on public genome databases of wild diploid sweetpotato (*I. trifida*)^20^ and morning glory (*I. nil*)^27^ showed that these two species also each have two *ZEP* genes (*I. trifida*: Itr_sc000013.1_g00031.1 and Itr_sc000013.1_g00027.1; *I. nil*: INIL11g18864.t1 and INIL11g18861.t1); Fig. 6a). In the sweetpotato genome, g1103.t1 and g1106.t1 were located at a distance of 16.4 kb in the same linkage group (Fig. 6b), and INIL11g18864.t1 and INIL11g18861.t1 were located at a distance of 20.9 kb in the same chromosome 11 of the morning glory genome. These genome structures support the idea that g1103.t1 and g1106.t1 are paralogous genes.

### Gene expression analysis for *ZEP* paralogs by quantitative real-time PCR

To confirm the results of RNA-seq, the expression patterns of g1103.t1 (*ZEP*), g1106.t1 (*ZEP*) and g16874.t1 (*PSY*) in BH and its white-fleshed mutants were analysed using quantitative real-time polymerase chain reaction (qRT-PCR) (Supplementary Table S9). The qRT-PCR results showed that the expression levels of g1103.t1 in WH2 and WH3 were much lower than that in BH. The expression levels of g1106.t1 and g16874.t1 in BH and its white-fleshed mutants were almost similar, which was also consistent with RNA-seq results. These results indicate the high reproducibility of the RNA-seq in this study.

Next, differences in the expression levels of g1103.t1, g1106.t1 and g16874.t1 were measured using storage roots at 11 weeks after planting and leaves at 7 weeks after planting from five yellow-fleshed cultivars and five white-fleshed cultivars (Table 1). The expression of g1103.t1 was detected in all yellow-fleshed cultivars and was higher in storage roots at 11 weeks after planting than in leaves at 7 weeks after planting. On the other hand, g1103.t1 expression was much lower than in all five white-fleshed cultivars. In three of the white-fleshed cultivars in particular, East Cape 4, Joy White and Lingnan 1, the expression of g1103.t1 was too low to detect. In contrast, g1106.t1 was similarly expressed in white- and yellow-fleshed cultivars. Expression levels of g1106.t1 were significantly higher in leaves at 7 weeks after planting than in storage roots at 11 weeks after planting. Significant difference in the expression level of g16874.t1 were not also detected between storage roots of yellow-fleshed cultivars and white-fleshed cultivars although g16874.t1 showed higher expression in BH than in WH2 and WH3 in RNA-seq analysis. This suggests that g16874.t1 is not involved in carotenoid accumulation in storage roots.

**Table 1:** Gene expression of g1103.t1 and g1106.t1 in storage roots (SRs) at 11 weeks after planting and leaves at 7 weeks after planting of 10 sweetpotato cultivars.

Cultivar^a^	Flesh colour	− ΔΔCt (g1103.t1)^c^		− ΔΔCt (g1106.t1)^c^		− ΔΔCt (g16874.t1)^c^	
		SR	Leaf	SR	Leaf	SR	Leaf
Beniharuka	Pale yellow	0	− 3.04	0	3.73	0	7.98
Benimasari	Pale yellow	0.36	− 2.46	0.54	2.95	− 0.25	8.13
Nongdahong	Pale yellow	0.30	− 1.30	0.51	5.74	0.34	9.22
Tamaotome	Yellow	0.51	− 1.61	0.97	4.76	1.43	7.96
Cavite	Yellow	− 1.21	− 2.45	1.35	4.96	2.28	8.68
Konamizuki	White	− 9.34	− 7.45	0.16	3.25	− 0.02	8.14
East Cape 4	White	< − 10	n.m	0.60	3.74	2.32	8.17
Joy White	White	< − 10	n.m	0.80	4.60	− 0.30	8.36
Lingnan 1	White	< − 10	n.m	− 0.21	3.79	0.60	7.50
Koganemasari	White	− 8.80	− 4.96	0.32	4.30	0.35	8.15
Mean^b^	Pale yellow & yellow	− 0.01 a	− 2.17 b	0.67 b	4.43 a	0.76 b	8.39 a
	White	–	–	0.33 b	3.94 a	0.59 b	8.06 a

^a^The flesh colours of all cultivars are shown in Supplementary Fig. S3.

^b^Mean values followed by the same letter in g1103.t1, g1106.t1 and g16874.t1 are not significantly different by Tukey’s honestly significant difference (HSD) test (*P* < 0.01).

^c^Relative expression values (− ΔΔCt) were calculated by the following formula: −((ΔCt in the target) − (ΔCt in SR of Beniharuka)). The exact relative expression values of samples with “n.m.” were not measurable because the Ct values of g1103.t1 were more than 30 cycles and amplification of primer dimers was observed.

## Discussion

In this study, transcriptome changes between a yellow-fleshed sweetpotato cultivar Beniharuka (BH) and its white-fleshed mutants (WH2 and WH3) were compared to reveal the key genes involved in the yellow pigmentation of the sweetpotato storage roots. We found that the gene expression of g1103.t1, which is considered to be a *ZEP* paralog, was considerably downregulated in WH2 and WH3 compared to BH. The results in this study provide new knowledge about the molecular mechanism of carotenoid accumulation in sweetpotato storage roots and may serve as a basis for the efficient breeding of yellow-fleshed cultivars.

ZEP, the enzyme in the biosynthetic pathway of the carotenoid metabolism, converts zeaxanthin to antheraxanthin and then converts antheraxanthin into violaxanthin by β-ring epoxidation^16,28^ (Fig. 1). Therefore, loss of *ZEP* function leads to zeaxanthin accumulation in the leaves of *A. thaliana*^29^ and *N. plumbaginifolia*^30^. Additionally, zeaxanthin was accumulated in potato tubers by suppressing *ZEP* expression, and the tubers showed a slightly deeper yellow colour^31^. Unique carotenoids in sweetpotato such as β-carotene 5,8;5′,8′-diepoxide and β-cryptoxanthin 5,8-epoxide are also synthesised by β-ring epoxidation of β-carotene and β-cryptoxanthin^7,9^. It was predicted that this epoxidation was regulated by a novel enzyme encoded by the *ZEP* paralog^7^. However, no such *ZEP* paralog has yet been identified in sweetpotato. In WH2 and WH3, zeaxanthin did not accumulate, despite the fact that the expression of g1103.t1, predicted as *ZEP,* was downregulated compared to BH (Fig. 3, Supplementary Table S9). This suggests that g1103.t1 is not associated with zeaxanthin epoxidation. Additionally, β-cryptoxanthin epoxides and β-carotene epoxides did not accumulate in WH2 and WH3, supporting the hypothesis that g1103.t1 is a *ZEP* paralog catalysing the epoxidation of β-carotene and β-cryptoxanthin.

The ZEP enzyme was encoded by a single gene in *A. thaliana*^29^ and *N. plumbaginifolia*^30^. However, phylogenetic analysis in this study indicated that sweetpotato had two genes, g1103.t1 and g1106.t1, with high homology to *ZEP* in other plants (Fig. 6a). Additionally, two close relatives of sweetpotato, *I. trifida* and *I. nil*, also had two genes each that showed high homology to *ZEP* and were considered to be paralogous. The two *ZEP* homologs in sweetpotato and *I. nil* are located close to each other in LG1 and Chr11, respectively, with the same strand (Fig. 6b). These genome structures imply that sweetpotato and *I. nil* obtained the *ZEP* paralog by duplication of the locus including the *ZEP* gene through evolution. Some plants can also synthesis unique carotenoids by duplication and neofunctionalisation of the genes involved in carotenoid synthesis^32^. Capsanthin and capsorubin are synthesised by capsanthin-capsorubin synthase (CCS) in only a few species (e.g., *Capsicum annuum* and *Lilium lancifolium*)^33^. Based on sequence similarity, it has been suggested that the *CCS* gene may have evolved from the lycopene cyclase (*LCY*) gene because of gene duplication^33,34^. Likewise, *ZEP* (g1103.t1-type ZEP) of sweetpotato would obtain a new function such as epoxidation of β-carotene or β-cryptoxanthin via gene duplication.

The expression of g1103.t1 was extremely low in all white-fleshed cultivars compared to that in yellow-fleshed cultivars (Table 1). The cultivars used in this study were genetically diverse. Nongdahong, Cavite, East Cape 4 and Lingnan 1 are exotic germplasms from China, the Philippines, Papua New Guinea and Taiwan, respectively, and the others are Japanese breeding cultivars. Thus, the relationship between yellow flesh and g1103.t1 expression level is not specific for BH and its white-fleshed mutants. Yellow-fleshed cultivars such as Benimasari and Tamaotome were found to contain sweetpotato carotenoids (i.e,. β-carotene 5,8;5′,8′-diepoxide, β-cryptoxanthin 5,8-epoxide, etc.) as did BH^4,6^. This suggests that the translated product of g1103.t1 is commonly involved in the synthesis of these carotenoids in yellow-fleshed cultivars.

The other *ZEP* paralog, g1106.t1, seemed to encode normal ZEP because the relative expression value of g1106.t1 was higher in leaves at 7 weeks after planting than in storage roots at 11 weeks after planting in all cultivars (Table 1). ZEP is the key enzyme for the xanthophyll cycle, which is the mechanism that protects photosynthetic tissues from photodamage^35^. Therefore, the expression level of the *ZEP* gene is usually higher in photosynthetic tissues than in non-photosynthetic tissues^36–38^. In contrast, the expression level of g1103.t1 was found to be higher in storage roots at 11 weeks after planting than in leaves at 7 weeks after planting in yellow-fleshed cultivars (Table 1). The function of the two *ZEP* paralogs may be separated; one is associated with the epoxidation of β-carotene and β-cryptoxanthin in roots and the other is associated with the epoxidation of zeaxanthin and antheraxanthin in leaves.

The transcription of genes related to carotenoid biosynthesis is known to be regulated by many different genetic factors^39^. In sweetpotato, it has been reported that *IbOr* promotes carotenoid accumulation by increasing gene expression in carotenoid biosynthesis^13,14^. In the present study, the expression level of g20467.t1 annotated as *Or* did not differ between BH and its white-fleshed mutants (Fig. 5). However, many DEGs other than g1103.t1 were detected in WH2 and WH3 (Fig. 4). Therefore, downregulation of g1103.t1 in WH2 and WH3 might be caused by any of the other DEGs. Furthermore, it has been reported for *Citrus* species that *ZEP* expression levels are regulated by differences in *cis*-elements among *ZEP* alleles^40,41^. In potato tubers, *ZEP* expression and flesh colour is related to the dosage of *ZEP* alleles^42,43^. Because sequence diversity in the locus of g1103.t1 among BH, WH2, WH3 and the other cultivars was not examined in this study, it is unclear whether the white flesh is caused by mutation in g1103.t1 itself or in any regulatory genes. In order to reveal the molecular mechanisms of carotenoid accumulation in sweetpotato, further study is necessary to identify the genetic factors that suppress g1103.t1 expression.

In this study, g1103.t1 was identified as a candidate gene involved in yellow colour in sweetpotato storage roots. Although we hypothesise that g1103.t1 regulates flesh colour through the epoxidation of β-carotene and β-cryptoxanthin, the function of the protein encoded by g1103.t1 remains unknown. In further study, the function of the g1103.t1 protein should be revealed by a molecular biological and biochemical approach.

## Methods

### Plant materials and growth conditions

A sweetpotato cultivar Beniharuka (BH), whose flesh colour is pale yellow, and two spontaneous white-fleshed mutants of BH (WH2 and WH3) were used in this experiment. Two white-fleshed storage roots obtained independently from different BH clones were vegetatively propagated and named WH2 and WH3, respectively. White fleshed mutation of these lines was stable through vegetative propagation of several years.

Stem cuttings of each line were transplanted into 5-L pots filled with vermiculite and grown in a greenhouse for 10 weeks. Following Tanaka et al. (2005)^44^, water and nutrition were supplied from the bottom of the pots using 1000-fold diluted Hyponex medium (N–P–K = 6.5–6–19; Hyponex Japan Co., Ltd, Osaka, Japan). After measuring the fresh weight of shoots, the number of storage roots, and the weight of total storage roots, International Commission on Illumination (CIE) l*a*b* values on the centre of cross-sections of storage roots were measured using a CM-2600d spectrophotometer (Konica Minolta, Inc., Tokyo, Japan). The storage roots including all internal tissues were then sliced and frozen in liquid nitrogen immediately. Samples were stored at − 80 °C until carotenoids and RNA were extracted. The experiment was carried out in triplicate using three plants (pots) for each line.

In order to study varietal differences in the expression of the candidate genes detected by RNA-seq, the stem cuttings of the five yellow-fleshed cultivars, Beniharuka, Benimasari, Nongdahong, Tamaotome and Cavite, and the five white-fleshed cultivars, Konamizuki, East Cape 4, Joy White, Lingnan 1 and Koganemasari, were cultivated as described above without replication (Supplementary Fig. S3). Unfolded leaves and storage roots were sampled at 7 and 11 weeks after planting, respectively, and frozen in liquid nitrogen immediately. These samples were also stored at -80 °C until RNA extraction.

### Evaluation of carotenoid composition

Carotenoid extraction from storage roots was performed following Ishiguro et al. (2010)^4^. Two grams of freeze-dried powder of storage roots was added to 6 mL acetone and mixed using a vortex mixer. The mixtures were centrifuged at 1500 *g* for 10 min. After a 2-mL aliquot of the supernatant was removed, 6 mL of acetone was added to the remainder. After re-extraction, 6 mL of the supernatant was taken and combined with the first extract (total 8 mL). The extract was then evaporated by a centrifugal concentrator, and the residue was redissolved in 1 mL tetrahydrofuran containing 0.1% butylated hydroxytoluene (BHT) and filtered through a 0.2-μm membrane filter.

Carotenoid compositions were detected by HPLC following the method described by Ishiguro et al. (2010)^4^. Relative carotenoid content was estimated from the ratio of the total peak area of each line to that of BH. Carotenoid composition was estimated using authentic reagents (β-carotene) and extracts from the Tamaotome cultivar (analysed by Ishiguro et al. 2010^4^) as standard.

### RNA extraction, library construction and RNA-seq

Total RNA in storage roots was extracted following the method described by Takahata et al. (2010)^45^. Total RNA in leaves was extracted using an RNeasy Plant Mini Kit (Qiagen, Hilden, Germany) according to the manufacturer’s protocol for qRT-PCR. The concentration and purity of extracted RNA were checked by SimpliNano (Biochrom Ltd., Cambridge, UK) and the degree of RNA degradation was checked by electrophoresis on 1.5% agarose gel.

Total RNA extracted from the storage roots of BH, WH2 and WH3 was used for the further step of RNA-seq library construction. RNA integrity confirmation (RNA Integrity Number > 8), library construction and RNA-seq were performed at Macrogen Japan Corp. (Kyoto, Japan). The cDNA library was constructed using a TruSeq Stranded mRNA LT Sample Prep Kit (Illumina, Inc., CA, USA) and sequenced using Illumina NovaSeq 6000.

### Analysis of RNA-seq data

Low-quality reads and Illumina adaptor sequences were removed by Trimmomatic (v0.36.6)^46^. The processed paired-end reads were aligned to the reference genome sequence of sweetpotato (cv Taizhong6) obtained from the Ipomoea Genome Hub (https://ipomoea-genome.org/)^19^ by HISAT2 (v2.1.0)^47^. The reads mapped to each gene were counted by featureCounts (v1.6.4)^48^, and the transcripts per million (TPM) value was calculated^49^. Differential gene expression between BH and WH2 or WH3 was analysed by edgeR (v3.24.3) and the logFC, average logCPM and FDR were calculated^50^. Genes with a |LogFC|≥ 2, average logCPM ≥ 1 and FDR ≤ 0.01 were considered DEGs.

Transcript sequences of sweetpotato were also obtained from the Ipomoea Genome Hub and annotated by Hayai-Annotation Plants (v1.0.2)^51^ with a nucleotide sequence identity > 40%, query coverage > 40% and e-value < 10^–6^. GO functional classification of DEGs was performed by WEGO (v2.0)^52^.

### Phylogenetic analysis of ZEP

The amino acid sequences deduced from g1103.t1, g1106.t1, g41700.t1 and g55667.t1 were used for phylogenetic analysis. Additionally, the amino acid sequences of ZEP in another eight plants were obtained from the National Centre for Biotechnology Information (NCBI) database. Using the ZEP sequences of *Arabidopsis thaliana* (GenBank: AAM13144.1) as query, TBLASTN searches were performed on genome databases of *Ipomoea trifida* (ITR_r1.0: https://sweetpotato-garden.kazusa.or.jp/index.html)^20^ and *Ipomoea nil* (Asagao_1.2: https://viewer.shigen.info/asagao/index.php)^27^, and the deduced amino acid sequences with high similarly to AAM13144.1 were also used. Multiple alignment was conducted by Clustal W^53^ and phylogenetic analysis was carried out using the neighbor-joining method^54^. Bootstrap analysis was performed with 1000 replications. The results were drawn using FigTree (v1.4.4) (https://tree.bio.ed.ac.uk/software/figtree/).

### qRT-PCR assay for *zeaxanthin epoxidase* genes

cDNA was synthesised from 0.5 μg of total RNA using a PrimeScript RT Reagent Kit with gDNA Eraser (Takara Bio Inc., Shiga, Japan). Primers for qRT-PCR were designed using specific sequences of g1103.t1 (forward: CGTTCCAACTCGTTTCCATCCTTC; reverse: CGTCTCGGGGCAGTTAATGATTCC), g1106.t1 (forward: CCCTGCATAGTTGGCTCTGT; reverse: CGCAAATCAGTCACGAAGAA) and g16874.t1 (forward: TGAAGCAGAACGAGGAGTAACC; reverse: GCAACAGGCAACATAAGCAAC). *Actin* (forward: TGTTAGCAACTGGGATGATATGG; reverse: GGATAGCACAGCCTGAATAGC)^55^ was used as a reference gene to normalise the gene expression of targets. qRT-PCR was conducted in 12.5 μL reaction volume using TB Green Premix Ex Taq II (Takara Bio Inc., Shiga, Japan) and the CFX Connect Real-Time PCR Detection System (Bio-Rad Laboratories, Inc., CA, USA). The temperature conditions for real-time qRT-PCR were as follows: 95℃ for 30 s, followed by 40 cycles of 95℃ for 5 s and 64℃ for 30 s (g1103.t1), and 95℃ for 30 s followed by 40 cycles of 95℃ for 5 s and 62℃ for 30 s (g1106.t1, g16874.t1 and *Actin*). Following the completion of amplification, a melting curve analysis was conducted to validate the uniformity of the amplification product. Data of samples amplified after 30 cycles that seemed to include nonspecific product were excluded from this analysis. The expression of each gene relative to the actin gene was determined by the ΔΔCt method^56^.

## Supplementary information

Supplementary Figures.

Supplementary Tables.

## Data Availability

RNA-Seq data are deposited in the Sequence Read Archive (DRA) of DNA Data Bank of Japan (DDBJ) under the accession number of DRA010350.
