# Author Correction: Comparative transcriptome analysis implied a *ZEP* paralog was a key gene involved in carotenoid accumulation in yellow-fleshed sweetpotato

**DOI:** 10.1038/s41598-021-98699-x

**Published:** 2021-09-20

**Authors:** Keisuke Suematsu, Masaru Tanaka, Rie Kurata, Yumi Kai

**Affiliations:** Kyushu Okinawa Agricultural Research Centre, National Agriculture and Food Research Organisation, 6651‑2 Yokoichi, Miyakonojo, Miyazaki 885‑0091 Japan

Correction to: *Scientific Reports* 10.1038/s41598-020-77293-7, published online 26 November 2020

The original version of this Article contained a repeated error in the Results section, under the subheading ‘Gene expression analysis for *ZEP* paralogs by quantitative real-time PCR’, in Table 1, and in the Methods section, under the subheading ‘qRT-PCR assay for *zeaxanthin epoxidase* genes’, where

“g16894.t1”

now reads:

“g16874.t1”

In addition, the primer sequence of Actin was incorrect in the Methods section, under the subheading ‘qRT-PCR assay for *zeaxanthin epoxidase* genes’, where

“*Actin* (forward: GTTCATTCAAGCCCACAGAG; reverse: TCCTTCCTTCTTCATCAATCTTC)^55^ was used as a reference gene to normalise the gene expression of targets.”

now reads:

“*Actin* (forward: TGTTAGCAACTGGGATGATATGG; reverse: GGATAGCACAGCCTGAATAGC)^55^ was used as a reference gene to normalise the gene expression of targets.”

The original Article has been corrected.

