# Supplementary material for: Comparative transcriptome analysis implied a ZEP paralog was a key gene involved in carotenoid accumulation in yellow-fleshed sweetpotato
Source: Sci Rep. 2020 Nov 26;10:20607. doi: 10.1038/s41598-020-77293-7 (PMC7693279; doi:10.1038/s41598-020-77293-7)
Supplement: Supplementary file 1 — Supplementary Figures. [file 41598_2020_77293_MOESM1_ESM.pdf]

Supplementary information

Comparative transcriptome analysis implied a *ZEP* paralog was a key gene involved in carotenoid accumulation in yellow-fleshed sweetpotato

Keisuke Suematsu[1*], Masaru Tanaka[1], Rie Kurata[1] and Yumi Kai[1]

[1] Kyushu Okinawa Agricultural Research Centre, National Agriculture and Food Research Organization, Yokoichi 6651-2 Miyakonojo, Miyazaki 885-0091, Japan

***Corresponding author:** Keisuke Suematsu

E-mail address: suematsuk578@affrc.go.jp

Supplementary Table S1. Mean values (n = 3) of shoot and storage root fresh weight, number of storage roots, dry matter and International Commission on Illumination (CIE) *l*a*b** values of storage roots in the cultivar Beniharuka (BH) and its two white-fleshed mutants WH2 and WH3.

Supplementary Table S2. Relative content (n = 3) of carotenoids in the storage roots of the cultivar Beniharuka (BH) and its two white-fleshed mutants WH2 and WH3.

Supplementary Table S3. Upregulated differentially expressed genes (DEGs) in WH2.

Supplementary Table S4. Upregulated DEGs in WH3.

Supplementary Table S5. Upregulated DEGs common to WH2 and WH3.

Supplementary Table S6. Downregulated DEGs in WH2.

Supplementary Table S7. Downregulated DEGs in WH3.

Supplementary Table S8. Downregulated DEGs common to WH2 and WH3.

Supplementary Table S9. Comparison of gene expression levels of g1103.t1, g1106.t1 and g16894.t1 between RNA-seq and quantitative real-time polymerase chain reaction (qRT-PCR).

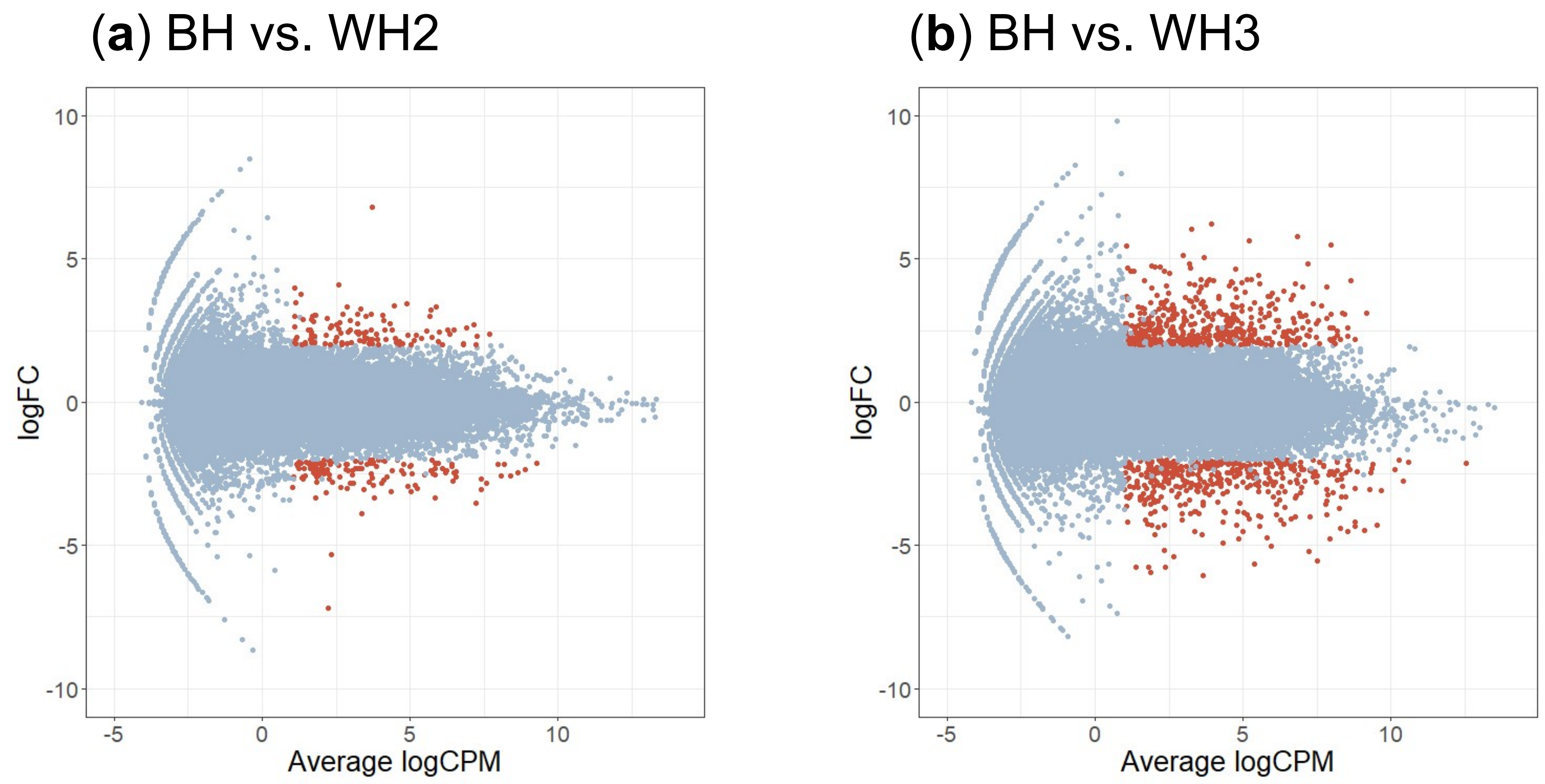

Supplementary Fig. S1. Relationship between gene expression level (average log2 counts per million, logCPM) and difference (logFC) in (a) BH vs. WH2 and (b) BH vs. WH3. Red dots indicate DEGs and grey dots indicate non-DEGs.

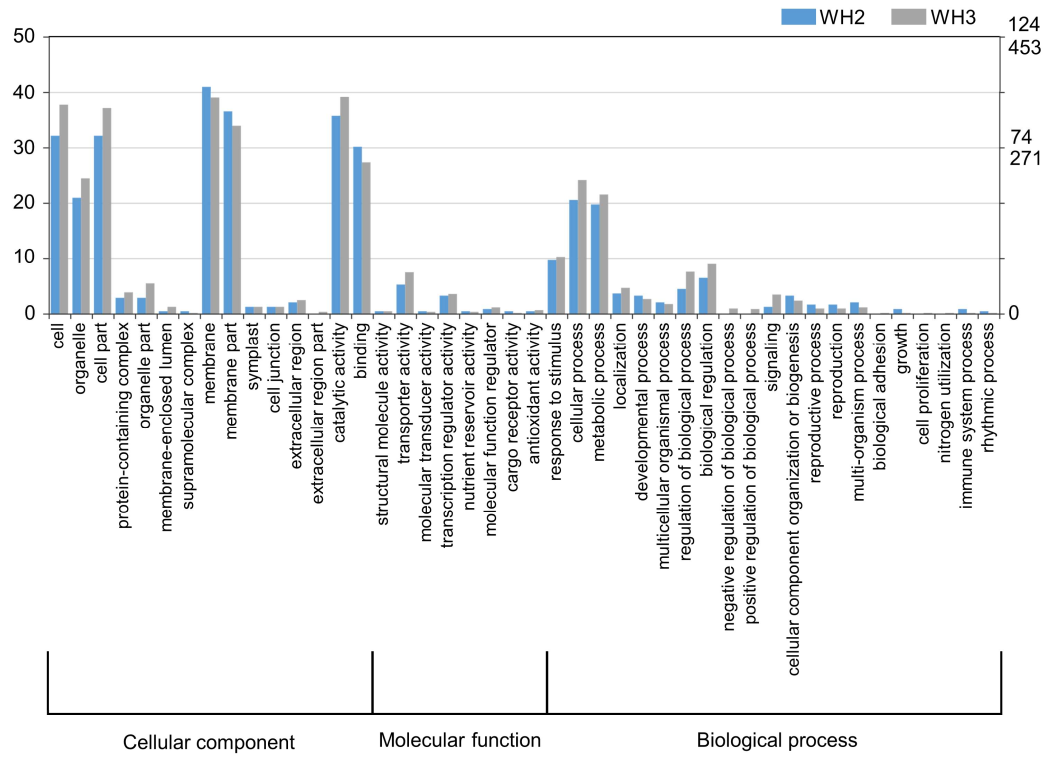

Supplementary Fig. S2. Gene ontology (GO) classification of DEGs in WH2 and WH3.

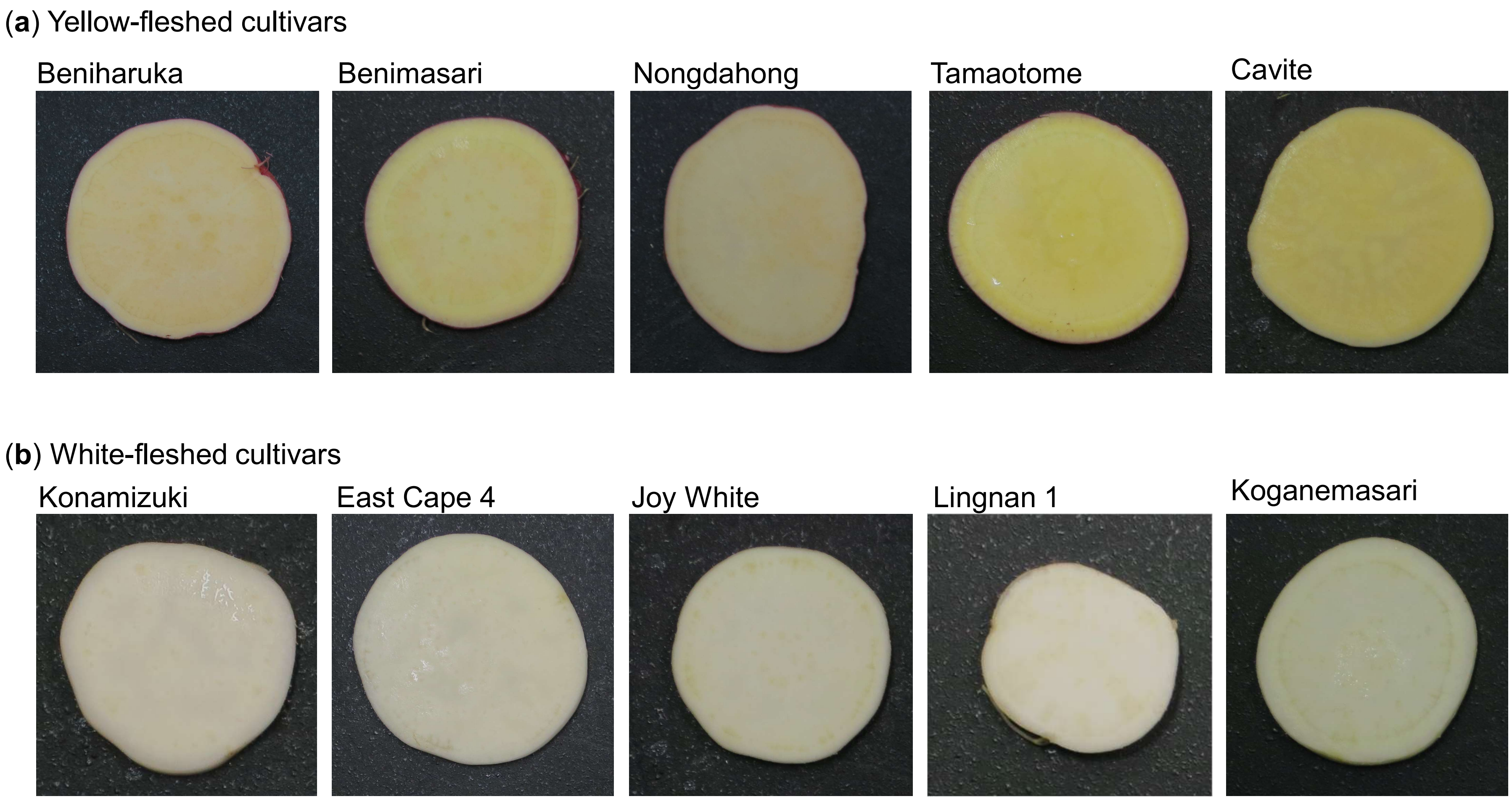

Supplementary Fig. S3. Cross-sections of storage roots of the (a) yellow-fleshed cultivars and (b) white-fleshed cultivars used in this study.
